# Effects of customer relationship management (CRM) strategies and socio-cognitive constructs on the physical activity of individuals with arthritis over time

**DOI:** 10.1371/journal.pone.0292692

**Published:** 2023-10-10

**Authors:** Fiona J. Newton, Tilahun N. Haregu, Joshua D. Newton, Robert Donovan, Ajay Mahal, Ruth Mackenzie-Stewart, Michael T. Ewing, Adrian Bauman, Karine E. Manera, Ben J. Smith

**Affiliations:** 1 Department of Marketing, Monash Business School, Monash University, Frankston, Victoria, Australia; 2 Nossal Institute for Global Health, Melbourne School of Population and Global Health University of Melbourne, Melbourne, Victoria, Australia; 3 Department of Marketing, Deakin Business School, Deakin University, Geelong, Victoria, Australia; 4 School of Human Sciences, University of Western Australia, Perth, Western Australia, Australia; 5 School of Psychology and Public Health, La Trobe University, Bundoora, Victoria, Australia; 6 Faculty of Business, Law & Arts, Southern Cross University, Gold Coast Campus, Bilinga, Queensland, Australia; 7 Distinguished visiting professor, Department of Marketing, University of Johannesburg, Johannesburg, South Africa; 8 School of Public Health, Level 6, The Charles Perkins Centre, University of Sydney, Sydney, New South Wales, Australia; 9 School of Public Health and Preventive Medicine, Monash University, Melbourne, Victoria, Australia; University of Minnesota School of Dentistry, UNITED STATES

## Abstract

**Background:**

Regular physical activity is important for arthritis self-management and could be promoted through tailoring community leisure and fitness centers’ customer-relationship management (CRM) strategies.

**Objectives:**

This study investigates the influence of two CRM strategies on individuals with arthritis reaching or maintaining two moderate-to-vigorous physical activity (MVPA) thresholds (≥150 and ≥45 minutes/week) from baseline-to-12 months and 12-to-24 months as well as mean changes in total minutes/week of MVPA. It also explores time-dependent variations in the influence of socio-cognitive variables on MVPA outcomes.

**Methods:**

Survey data from 374 participants with arthritis in a two-year randomized controlled trial (control versus two CRM strategies: Incentive^Only^ and Incentive^+Support^) were used. Participants reported measures of physical activity participation, socio-cognitive decision-making, mental and physical wellbeing, friendship, community connectedness, sense of trust in others, and demographics.

**Findings/discussion:**

Receiving the Incentive^+Support^ CRM strategy (versus control) increased participants’ likelihood of reaching/maintaining both physical activity thresholds from 12-to-24 months (≥150 MVPA minutes/week, p < .001; ≥45 MVPA minutes/week, p < .032) but not from baseline-to-12 months. However, receiving the Incentive^Only^ CRM strategy (versus control) did not predict reaching/maintaining these thresholds. Importantly, socio-cognitive decision-making variables’ influence on reaching/maintaining these MVPA thresholds varied over time, suggesting CRM strategies may require further tailoring based on time-specific profiles. Perhaps because of new facility induced excitement, the mean change in total MVPA minutes/week for the control group significantly increased (26.8 minute/week, p = .014, 95% CI [5.5, 48.0]) from baseline-to-12 months, but subsequently declined by 11.4 minute/week from 12-to-24 months (p = .296, 95% CI [-32.7, 9.9]). Mean changes in total MVPA minutes/week were non-significant for those receiving Incentive^Only^ content but significant for those receiving Incentive^+Support^ content: baseline-to-12 months (38.2 minute/week increase, p = .023, 95% CI [4.9, 71.4]) and baseline-to-24-months (45.9 minute/week increase, p = .007, 95% CI [12.7, 79.1]).

## Introduction

Regular physical activity is an important component in self-managing common arthritic conditions including osteoarthritis, rheumatoid arthritis, and psoriatic arthritis [1, 2], particularly as a means of reducing joint stiffness and pain as well as improving overall functioning, quality-of-life, and mood [3–5]. The potential benefits that can accrue from increasing physical activity have led to numerous self-management programs, including: web-based interventions that combine exercise with advice and other forms of support [6–8]; interactive digital interventions that include knowledge building around physical activity programs and mechanisms to set daily reminders and/or input personal data [9–11]; time-limited programs that individuals complete in their own homes [12]; and guided time-limited programs with face-to-face or telehealth input from allied healthcare professionals [13, 14].

What is less evident in extant literature is the potential for community leisure and fitness centers to also play a role in promoting and encouraging regular physical activity among typically inactive people with arthritis. The protracted nature of arthritic conditions means those impacted are likely to require uninterrupted access to physical activity resources for extended periods of time [2]. Multi-purpose leisure, aquatic and fitness centers are well-positioned to meet this need by providing year-round access to physical activity resources without factors such as weather and concerns about safety acting as barriers to engagement. Another, albeit underexamined, pathway for encouraging ongoing physical activity among typically inactive people with an arthritic condition is through the customer relationship management (CRM) systems of these facilities.

In CRM systems, organizations tailor their marketing communications and promotions using data they have collected and collated on current and prospective customers [15]. Research suggests CRM systems have the potential to generate and sustain patronage across time and deliver enhanced customer experiences [16] in a range of contexts, including healthcare services [17, 18] and fitness centers [19–21]. A key reason for such successes, and one pertinent to the current context, is that CRM systems enable organizations to use the data they have collated to strategically tailor their messaging and incentives to garner customer interest in, and engagement with, their offerings [22–24] and ultimately co-create value for system recipients [25]. This process of value-exchange requires a deep understanding of those being targeted with CRM strategies (and by extension program content) so that a persuasive value proposition (i.e., value statement) can be presented to them [25]. Such knowledge is key to harnessing CRM program content to promote health-related behaviors [26].

In examining the efficacy of using CRM program content to motivate physical activity it is important to understand the socio-cognitive characteristics of those being targeted, as constructs from socio-cognitive theories (e.g., the Theory of Planned Behavior and associated extensions) have been shown to explain why some individuals are more likely to enact physical activity behaviors than others [27, 28]. Understanding whether the influence of these socio-cognitive constructs on physical activity levels changes across time is also important, not only for informing the broader strategic design of future CRM program content but also for guiding CRM managers in the type of data they should collect to help develop suitably tailored CRM elements.

In this study, we draw on the Monitoring and Observing the Value of Exercise (MOVE) two-year randomized control trial (RCT; 2014–2016), which sought to encourage regular physical activity among those that are typically inactive (see Newton et al. [29] for the trial protocol), to better understand the profiles of individuals with arthritis who reached or maintained two distinct thresholds of moderate to vigorous physical activity (MVPA) per week across specific timepoints during the trial. First, we profiled those who reached or maintained the World Health Organization’s (WHO) [30] recommendation of at least 150 minutes per week (≥150 min/wk) of MVPA between entry into the MOVE trial and 12 months post-baseline, and again between 12- and 24-months post-baseline. Second, drawing on research suggesting individuals with various forms of arthritis can find it difficult to achieve this upper threshold of ≥150 min/wk of MVPA [31, 32], we also profiled those reaching or maintaining an intermediate threshold of at least 45 min/wk of MVPA across the same timepoints. This intermediate threshold was selected because prior research suggests therapeutic benefits can accrue from lower levels of physical activity [33, 34]. Indeed, 45 min/wk of MVPA has been found predictive of improvements in physical functioning over a two-year period among individuals with osteoarthritis of the lower extremities (i.e., hip, knee, foot, or ankle) who have low levels of function at baseline [35]. As part of this profiling, we examined whether those individuals with arthritis who received either of the CRM strategies that formed part of the MOVE trial were more likely than those in the control group to reach/maintain the upper and intermediate thresholds of MVPA/wk across the trial’s three timepoints. Finally, we examined whether there were significant mean changes in total MVPA min/wk among those who received each type of CRM strategy as well as the control group.

The findings from this study add to the emerging literature on the use CRM strategies to co-create health-related benefits for system recipients. The findings also have implications for multi-purpose leisure, aquatic and fitness center management teams, who should consider gathering and using specific socio-cognitive information from the recipients of CRM strategies to create persuasive value propositions. Regularly updating this information may be necessary to maximize the influence of CRM strategies on MVPA levels.

## Materials and methods

### MOVE trial setting

The MOVE trial was designed to encourage physically inactive residents of the City of Frankston in Victoria, Australia, to engage in physical activity, including utilizing the new Peninsula Aquatic Recreation Centre (PARC) located adjacent to the central business district of the city. The PARC facility, developed at a cost of AUD$49.7 million, was designed to meet a diverse range of resident needs and includes a 50-metre indoor lap pool, warm water pool, learn to swim pools, an aquatic playground, gymnasium, group exercise rooms, as well as a spa, sauna and wellness therapies center.

### MOVE trial design

The MOVE trial collected data across three timepoints: baseline (prior to participant randomization), 12 months post-baseline, and 24 months post-baseline. The trial was undertaken with the cooperation of the PARC facility, received institutional approval from the Monash University Human Research Ethics Committee (Project IDs: CF14/1148–2014000497 and CF14/2059–2014001074), and is registered with the Australian New Zealand Clinical Trials Registry (Trial ID: ACTRN126150000 12572).

### MOVE trial participants and recruitment

Residents of the City of Frankston aged 18–70 years were eligible to participate in the MOVE trial if they did not, at the time of recruitment, attend a leisure/exercise facility 3 or more times per week and were classified as being typically physically inactive using Milton et al.’s [36] criterion of undertaking <5 occasions of 30 minutes or more of physical activity per week. Residents were excluded from the study if they were unable to walk independently, had poor English skills, had already purchased a PARC membership at the time of recruitment, or could not provide telephone or postal contact information. Recruitment primarily took place via telephone calls to a random sample of Frankston City Council residents with numbers listed in the Electronic White Pages directory for the area. Once contact was made (each telephone number was called 4–6 times), a computer-generated random ordering of persons in the household was used to determine who would be invited to undertake the MOVE trial screening questions. If this person was ineligible, they were asked if another person in the household could be screened for eligibility, and if so, the process was repeated. If an eligible person was identified, the trial explanatory statement was read to them and they were asked to provide verbal consent. A small proportion of the sample (5%) were recruited via face-to-face interviews in community venues (e.g., shopping centers) within socioeconomically disadvantaged areas. Three authors who managed the study field work (JN, RMS, BJS) had access to identifying information about study participants in order to conduct intervention delivery and follow-up measures.

### Current study participants

In this study, we draw on data from 374 individuals enrolled into the MOVE trial who self-reported having an arthritic condition at the baseline data collection point.

### MOVE trial CRM programs

Once participants had completed the baseline survey, electronic random number generation was used to randomize them on an individual basis (undertaken by author BJS), after which sequential ordering was used to allocate them to the control group or one of the two CRM strategies (undertaken by author JDN). The participants were not blinded to their group. The control group received no CRM related materials; they were, however, exposed to public promotions of the PARC facility (e.g., public billboards, local newspaper articles about the facility opening, and resident letters distributed by Frankston City Council).

Drawing on customer relationship methods [37] and social marketing principles [38], two CRM strategies were trialed. CRM Strategy 1 (Incentive^Only^) focused on encouraging uptake of physical activity through the provision of a voucher for one free trial visit to use the swimming pools or gymnasium at the PARC facility and an information pack about the facility. CRM Strategy 2 (Incentive^+Support^) included the voucher and information pack as well as ongoing supportive interactions with PARC in the form of a follow-up telephone call in the first 6 months to encourage trialing of the Centre, PARC-branded supportive messaging, and customer relationship materials. These included hand-written birthday and Christmas cards encouraging participants to achieve their physical activity goals as well as quarterly mailed newsletters designed to encourage physical activity by providing tips on how to overcome common barriers to engaging in physical activity. These types of supportive messaging were selected for trialing because of the ease with which they could be integrated into existing CRM systems and their low ongoing running costs.

### Measurement

The primary outcome measure for this study is physical activity, which was assessed using the Exercise Recreation and Sport Survey (ERASS) [39]. ERASS has concurrent validity to established population measures of total physical activity and provides a measure of the frequency and duration of organized and non-organized leisure activities undertaken in the past two weeks [39]. It has been used to compute minutes of MVPA per week and to subsequently categorize participants as reaching/maintaining the WHO’s [30] recommended ≥150 min/wk of MVPA threshold or our intermediate threshold of ≥45 min/wk of MVPA.

To develop a profile of those who did (or did not) reach/maintain these two MVPA thresholds, we drew on single-item measures of social and cognitive determinants of physical activity aligned with the Theory of Planned Behavior framework [40–45]. Each construct was assessed using a five-point Likert scale ranging from strongly disagree to strongly agree, such that agree responses were associated with higher levels of the construct. Specifically, participants reported their level of agreement in relation to: intending to engage in physical activity [40]; their attitude toward physical activity [41, 42]; having important others who would approve if they exercised regularly (subjective norm) [42]; having the ability to exercise if they wanted to (self-efficacy) [43]; feeling regretful if they did not exercise regularly (anticipated regret or inaction regret) [44], and having developed an action plan for physical activity [45]. More detail on these constructs is provided in S1 Appendix.

To assess the influence of health-related factors on physical activity, we drew on the single item global self-rated mental health measure [46] to capture emotional wellbeing and role functioning, and the single item global quality-of-life measure [47] to assess perceived physical health. The Functional Comorbidity Index (FCI) was used to measure chronic disease status (e.g., heart disease, diabetes, arthritis) [48]. Participants were also asked whether their arthritis limited their physical activity (yes/no) and whether they participated in ‘organized physical activity’ (yes/no). The six-item Friendship Scale was used to assess feelings of being supported and not socially isolated [49], while perceived community connectedness and sense of trust in others were assessed using two items taken from the Australian Unity Wellbeing Index [50]. Demographic details included gender, age, educational attainment, employment status, and household income.

The trial measures were administered at each data collection timepoint by a trained team via computer assisted telephone interviews. The interviews took approximately 20 minutes and the interviewers were blind to participant group allocation.

### Data analysis

Among MOVE trial participants reporting an arthritic condition at baseline, 90 were lost to follow-up at 12 months with 109 lost at the 24-month data collection point. Mann-Whitney test and chi-square tests indicated that those who were lost to follow-up did not differ from those retained at 12 months post-baseline and 24 months post-baseline in terms of their physical activity levels (the primary outcome variable), socio-demographic, and socio-cognitive variables at baseline. In line with Bell et al. [51], the cases were considered as missing at random with respect to physical activity and the Last Observation Carried Forward (LOCF) method was used to replace missing values for physical activity levels at 12 months and 24 months. That is, missing values were replaced with the last observed value for that construct. For example, if the last observed value for physical activity was at 12-months, this value was carried forward to replace the missing physical activity value at the 24-month timepoint. Prior to analysis, the constructs associated with social cognitive decision making around physical activity were dichotomized (agree vs. neutral/disagree). As noted above, agree responses are associated with higher levels of the construct. The proportions of participants in each category were then calculated.

The data analysis plan was developed at two levels of specificity: all participants with arthritis and those with arthritis by group allocation (i.e., control group and the two CRM strategy groups: Incentive^Only^ and Incentive^+Support^). At each level of specificity, comparisons of the control with the two CRM groups were undertaken. The socio-demographic and socio-cognitive characteristics are described using means (standard deviations) and proportions. Given the exploratory nature of this research and that we were not engaging in hypothesis testing, we used a stepwise logistic regression model with backward elimination to examine baseline predictors of individuals who reached or maintained (i) ≥150 min/wk and (ii) ≥45 min/wk of MVPA from baseline-to-12 months post-baseline. This approach has been used previously in the context of physical activity and health [52, 53] and has the advantage over forward stepwise methods in reducing the risk of making a Type II error, since it is less likely ‘to exclude predictors involved in suppressor effects’ [54 p227]. A similar analysis was used to examine 12-month predictors of individuals who reached or maintained ≥150 min/wk and ≥45 min/wk of MVPA/wk from 12-to-24 months post-baseline. Data pertaining to the socio-demographic and socio-cognitive characteristics of participants along with their participation in organized physical activity were included in the initial models. In each of these analyses, group allocation (i.e., control, Incentive^Only^ and Incentive^+Support^) was included as a covariate. This was done to assess whether the type of CRM strategy influenced physical activity levels. Predictors with p<0.1 were included in the final model, with p < .05 considered statistically significant. Next, we undertook analyses using mixed-effects models to examine the changes in total minutes of MVPA per week across the three MOVE trial data collection points among all participants with arthritis and by allocated group. Analyses were undertaken using Stata 16.0.

## Results

### Characteristics of the study population

Details of the baseline characteristics of participants are shown in Table 1. A total of 374 participants who reported an arthritic condition at baseline were included in this analysis; the mean age was 58.8 (SD = 9.4) years with 69% (n = 258) being female (Table 1). At the time of baseline assessment (2014), 33.7% of participants reported being in full-time employment and 80% had an annual household income of less than AU$80,000 in the fiscal year prior to the baseline assessment. At this baseline timepoint, 51.9% of participants reported that arthritis limited their physical activity levels and only 13.1% reported having no other comorbid condition.

**Table 1:** Demographic and health characteristics among individuals with arthritis upon entry into the MOVE trial—Baseline (n = 374).

Characteristic^†^	n	%
Group allocation		
Control—no CRM materials	155	41.4
CRM Incentive^Only^	118	31.6
CRM Incentive^+Support^	101	27.0
Gender		
Male	110	29.4
Female	258	69.0
Age in years: mean (SD)	58.8 (9.4)	
Educational status		
High school or less	153	40.9
Vocational qualification	144	38.5
University degree	75	20.1
Employment status		
Full-time employed	126	33.7
Part-time employed	75	20.1
Retired/Other	167	44.6
Household income (AU$)		
≤39,999	143	38.2
40,000 to 79,999	133	35.6
≥80,000	75	20.1
Functional Comorbidity Index-excludes arthritis		
None	49	13.1
One	105	28.1
Two	111	29.7
Three	74	19.8
≥Four	35	9.4
Arthritis limit activity at baseline		
Yes	194	51.9
No	180	48.1

^†^Not all counts total n = 374 because of missing data. SD = Standard deviation.

### Psycho-social characteristic of participants with arthritis at baseline and 12 months post-baseline

The description of socio-cognitive constructs in Table 2 focuses on two timepoints (baseline and 12-months post baseline) as data collected at these points are central to our subsequent multiple regression analyses examining predictors of the two MVPA min/wk thresholds from baseline-to-12 months post-baseline and from 12-to-24 months post-baseline. Given socio-cognitive data collected at 24-months post-baseline are not included in any analyses, they are not reported in Table 2. The majority of participants reported affirmative intentions to engage in physical activity (baseline = 82.6%; 12 months = 80.5%) and favorable attitudes towards physical activity (baseline = 75.7%; 12 months = 78.9%; see Table 2). Similarly, with respect to subjective norm, the majority agreed that those important to them would approve of them regularly engaging in physical activity (baseline = 93.6%; 12 months = 93.1%). Likewise, the majority reported having sufficient self-efficacy to engage in physical activity if they wanted to (baseline = 87.7%; 12 months = 84.5%), and that they would experience anticipated regret if they did not do so (baseline = 86.9%; 12 months = 87.7%). However, looking across the two timepoints, only around 5 in 10 agreed they had an action plan to engage in physical activity (baseline = 52.7%; 12 months = 56.7%) and around 4 in 10 had neutral/dissatisfied feelings about being part of the local community (baseline = 44.4%; 12 months = 44.7%). Similarly, around 5 in 10 reported neutral/dissatisfied feelings about whether most people could be trusted (baseline = 56.2%; 12 months = 52.1%). In terms of health, less than 2 in 10 reported fair-to-poor mental health (baseline = 15.8%; 12 months = 14.7%) and around 4 in 10 participants reported having fair-to-poor physical health (baseline = 41.2%; 12 months = 41.2%). In all, participation in organized physical activity was low (baseline = 17.4%; 12 months = 15.0%).

**Table 2:** Description of socio-cognitive constructs among all participants with arthritis (n = 374).

Construct	Baseline		12 months^†^	
	n	%	n	%
Intention				
Disagree/Neutral	62	16.6	72	19.3
Agree	309	82.6	301	80.5
Attitude				
Disagree/Neutral	90	24.1	79	21.1
Agree	283	75.7	295	78.9
Subjective norm				
Disagree/Neutral	22	5.9	24	6.4
Agree	350	93.6	348	93.1
Self-efficacy				
Disagree/Neutral	44	11.8	56	15.0
Agree	328	87.7	316	84.5
Anticipated regret				
Disagree/Neutral	48	12.8	44	11.8
Agree	325	86.9	328	87.7
Action planning				
Disagree/Neutral	176	47.1	162	43.3
Agree	197	52.7	212	56.7
Feeling part of community				
Neutral or dissatisfied	166	44.4	167	44.7
Moderately satisfied	99	26.5	83	22.2
Highly satisfied	108	28.9	123	32.9
Most people can be trusted				
Neutral or dissatisfied	210	56.2	195	52.1
Moderately satisfied	102	27.3	98	26.2
Highly satisfied	59	15.8	80	21.4
Friendship scale (Mean, SD)	26.8 (3.9)		26.9 (3.8)	
Perceived mental health				
Excellent to very good	194	51.9	202	54.0
Good	121	32.4	115	30.8
Fair-to-poor	59	15.8	55	14.7
Perceived physical health				
Excellent to very good	84	22.5	95	25.4
Good	135	36.1	124	33.2
Fair-to-poor	154	41.2	154	41.2
Participated in organized physical activity				
Yes	65	17.4	56	15.0
No	309	82.6	318	85.0

^†^Last observation carried forward (LOCF) replacement was used for participants who dropped out of the MOVE trial at the 12-month post-baseline data collection point. SD = Standard deviation.

### Predictors of reaching or maintaining 150 min/wk of MVPA

#### Baseline-to-12 months post-baseline

Table 3 shows two significant predictors increased the likelihood of reaching/maintaining the WHO upper threshold. Specifically, those who participated in some form of organized physical activity at baseline (OR = 3.82, 95% CI [2.10, 6.96]) and those who agreed that they intended to engage in physical activity at baseline (OR = 2.34, 95% CI [1.08, 5.08]) were more likely to have reached/maintained ≥150 min/wk of MVPA at 12 months than their respective counterparts. Two significant predictors decreased the likelihood of reaching/maintaining the WHO upper threshold; being female (OR = 0.48, 95% CI [0.28, 0.81]) and those with lower perceived physical health (OR = 0.49, 95% CI [0.29, 0.85]) were less likely to have met this threshold at 12 months. Notably, neither CRM strategy (i.e., Incentive^Only^ and Incentive^+Support^) significantly predicted reaching/maintaining the ≥150 min/wk of MVPA threshold at 12 months post-baseline when compared to the control group.

**Table 3:** Predictors of reaching/maintaining ≥150 min/wk of MVPA.

Construct		OR	95% CI		p value
**Baseline-to-12 months** ^†^					
Group allocation (Ref: Control)	CRM Incentive^Only^	1.10	0.62	1.97	.738
	CRM Incentive^+Support^	1.61	0.90	2.89	.110
Gender (Ref: Male)	Female	**0.48**	**0.28**	**0.81**	**.006**
Employment (Ref: Full-time)	Part-time	1.08	0.53	2.20	.826
	Retired/Other	1.72	0.99	3.01	.056
Organized PA (Ref: No)	Yes	**3.82**	**2.10**	**6.96**	**< .001**
Intention (Ref: Disagree/Neutral)	Agree	**2.34**	**1.08**	**5.08**	**.032**
Perceived physical health (Ref: Excellent to very good)	Good/fair-to-poor	**0.49**	**0.29**	**0.85**	**.012**
**12-to-24 months** ^†^					
Group allocation (Ref: Control)	CRM Incentive^Only^	1.74	0.97	3.13	.064
	CRM Incentive^+Support^	**3.13**	**1.70**	**5.77**	**< .001**
Gender (Ref: Male)	Female	0.60	0.35	1.02	.061
Education (Ref: High school or less)	TAFE certificate	1.70	0.95	3.01	.072
	University degree	**2.26**	**1.17**	**4.36**	**.015**
Employment^††^ (Ref: Full-time)	Part-time	1.37	0.65	2.88	.410
	Retired/Other	1.54	0.85	2.78	.152
Organized PA^††^ (Ref: No)	Yes	**3.19**	**1.67**	**6.07**	**< .001**
Attitude^††^ (Ref: Disagree/Neutral)	Agree	**3.45**	**1.63**	**7.30**	**.001**
Functional Comorbidity Index-excl arthritis^†^ (continuous)		0.85	0.70	1.05	.132

^†^Final model of a stepwise logistic regression model with backward elimination. Predictors with p<0.1 were included in the final model, with p < .05 considered statistically significant. The latter are printed in bold.

^**††**^ Data from the 12-month post-baseline data collection point.

#### 12-to-24 months post-baseline

Next, to explore potential changes in predictors of reaching/maintaining the ≥150 min/wk of MVPA threshold over time, we used socio-cognitive data collected at the 12-month timepoint to identify significant predictors of this threshold at 24 months post-baseline. As outlined in Table 3, four significant predictors were identified. In line with the baseline-to-12-month data, those who participated in some form of organized physical activity at 12 months post-baseline were more likely to have reached/maintained ≥150 min/wk of MVPA at 24 months post-baseline than their respective counterparts (OR = 3.19, 95% CI [1.67, 6.07]). Interestingly, compared to the control group, those who received the Incentive^+Support^ CRM strategy demonstrated a higher likelihood of reaching/maintaining ≥150 min/wk of MVPA at 24 months post-baseline (OR = 3.13, 95% CI [1.70, 5.77]). Reaching or maintaining the ≥150 min/wk threshold at 24 months post-baseline was also more likely among those reporting a positive attitude towards physical activity (OR = 3.45, 95% CI [1.63, 7.30]) and those with a university degree (OR = 2.26, 95% CI [1.17, 4.36]) relative to those with only a primary/high school education.

### Predictors of reaching or maintaining at least 45 min/wk of MVPA

#### Baseline-to-12 months post-baseline

The results from Table 4 indicate that those involved in some form of organized physical activity at baseline (OR = 2.62, 95% CI [1.32, 5.20]), agreed that they intended to engage in physical activity (OR = 2.66, 95% CI [1.29, 5.46]), expressed that they would experience regret if they did not engage in physical activity (OR = 2.38, 95% CI [1.05, 5.39]), and reported stronger friendship connections (OR = 1.07, 95% CI [1.01, 1.13]) were more likely to have reached/maintained the intermediate threshold of ≥45min/wk of MVPA at 12 months post-baseline. Similarly, those with an employment classification of ‘retired/other’ at baseline were more likely to achieve this threshold relative to those in fulltime employment (OR = 1.74, 95% CI [1.02, 2.98]). However, females were less likely than their male counterparts to have reached/maintained this intermediate threshold by 12 months post-baseline (OR = 0.49, 95% CI [0.29, 0.84]). Consistent with the findings regarding the upper threshold of ≥150 min/wk of MVPA, when compared to the control group, neither CRM strategy (i.e., Incentive^Only^ and Incentive^+Support^) were predictive for the intermediate threshold of ≥45 min/wk of MVPA at 12 months post-baseline.

**Table 4:** Predictors of reaching or maintaining ≥45 min/wk of MVPA.

Construct		OR	95% CI		p value
**Baseline-to-12 months**					
Group (Ref: Control)	CRM Incentive^Only^	0.61	0.36	1.04	.071
	CRM Incentive^+Support^	1.00	0.57	1.75	.991
Gender (Ref: Male)	Female	**0.49**	**0.29**	**0.84**	**.009**
Employment (Ref: Full-time)	Part-time	0.82	0.43	1.55	.542
	Retired/Other	**1.74**	**1.02**	**2.98**	**.043**
Organized PA (Ref: No)	Yes	**2.62**	**1.32**	**5.20**	**.006**
Intention (Ref: Disagree/neutral)	Agree	**2.66**	**1.29**	**5.46**	**.008**
Subjective norm (Ref: Disagree/neutral)	Agree	0.40	0.14	1.15	.089
Anticipated regret (Ref: Disagree/neutral)	Agree	**2.38**	**1.05**	**5.39**	**.038**
Arthritis limit activity (Ref: No)	Yes	1.46	0.91	2.33	.114
Friendship scale for social isolation		**1.07**	**1.01**	**1.13**	**.024**
**12-to-24 months**					
Group (Ref: Control)	CRM Incentive^Only^	1.15	0.58	2.29	.686
	CRM Incentive^+Support^	**2.29**	**1.07**	**4.89**	**.032**
Employment^†^ (Ref: Full-time)	Part-time	1.33	0.51	3.46	.557
	Retired/Other	1.58	0.71	3.52	.261
Annual income^†††^ (AU$) (ref: <40,000)	40,000–79,999	0.99	0.50	1.95	.976
	≥80,000	2.63	1.00	6.96	.051
Attitude^††^ (Ref: Disagree/neutral)	Agree	**3.62**	**1.68**	**7.80**	**.001**
Action planning^††^ (Ref: Disagree/neutral)	Agree	**2.20**	**1.20**	**4.03**	**.011**
Functional Comorbidity Index-excl arthritis^††^		0.85	0.64	1.13	.262
Arthritis limit activity^††^ (Ref: No)	Yes	0.74	0.38	1.44	.377

^†^Final model of a stepwise logistic regression model with backward elimination. Predictors with p<0.1 were included in the final model, with p < .05 considered statistically significant. The latter are printed in bold.

^††^ Data from the 12-month post-baseline data collection point.

^†††^As income data was not collected at 12-months post-baseline, the baseline data was used.

#### 12-to-24 months post-baseline

To further examine changes in the predictive utility of socio-cognitive variables over time, we drew on data collected at the 12-month timepoint to identify significant predictors of reaching/maintaining the intermediate physical activity threshold ≥45 min/wk of MVPA at 24 months post-baseline. As outlined in Table 4, a somewhat different pattern emerged. Participants who were more likely to reach/maintain this intermediate threshold at 24 months post-baseline exhibited three key factors: reporting a positive attitude towards physical activity (OR = 3.62, 95% CI [1.68, 7.80]), having an action plan (OR = 2.20, 95% CI [1.20, 4.03]), and being a recipient of the Incentive^+Support^ CRM strategy (relative to the control group; OR = 2.29, 95% CI [1.07, 4.89]).

### Mean changes in total MVPA min/wk by group allocation

**Fig 1:**
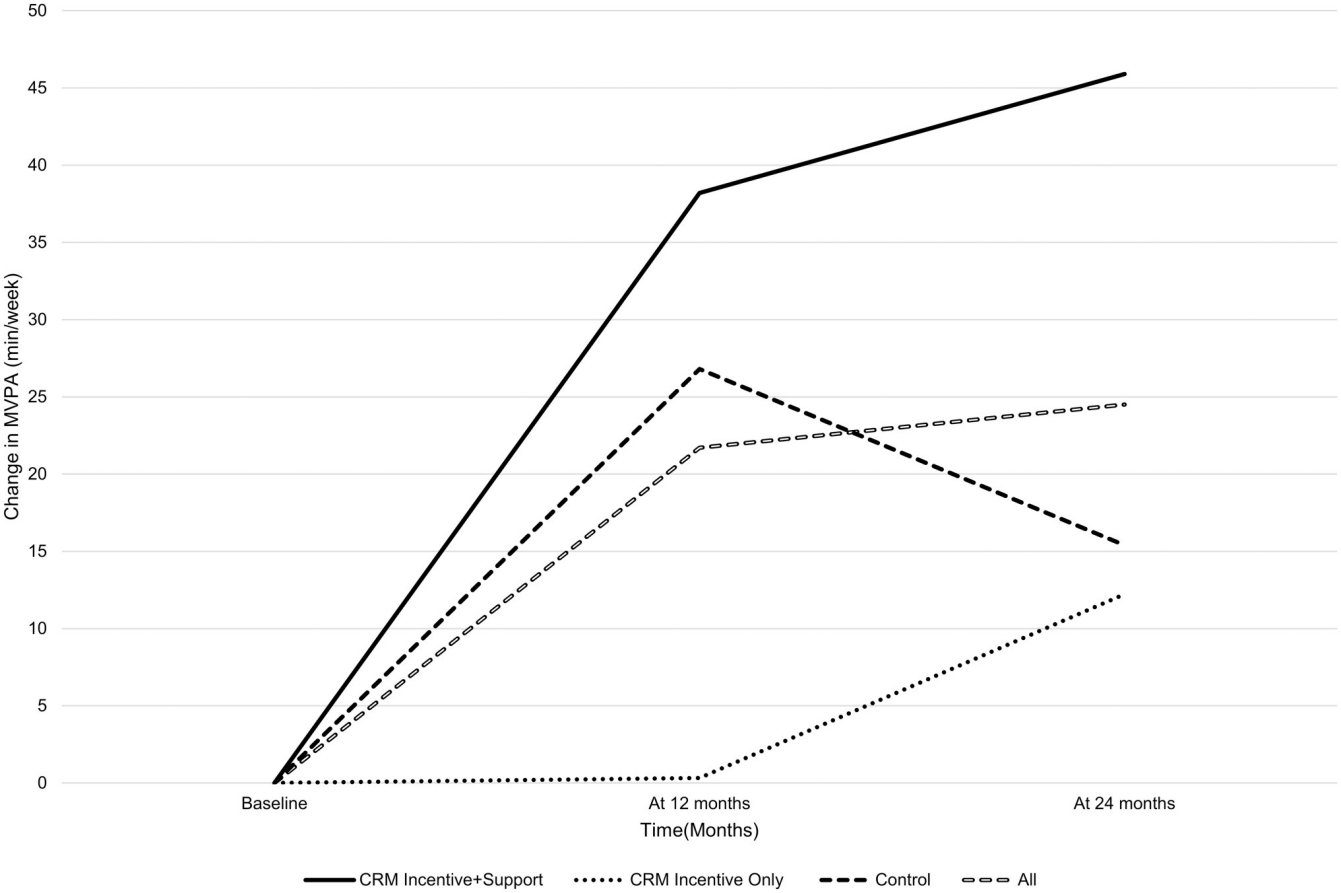
Additional increase in total minutes/week of MVPA by group.

Finally, we used mixed-effects models to examine mean changes in total min/wk of MVPA across three timepoints (baseline-to-24 months, baseline-to-12-months, and 12-to-24 months) among all participants with arthritis and separately for each type of CRM strategy and for the control group. As outlined in Table 5, when examined as a whole cohort, there was a significant mean increase of 24.5 min/wk of MVPA from baseline-to-24 months (p = .006, 95% CI [7.2, 41.9]). Across the full 24 months, there was a significant mean increase in MVPA of 21.7 min/wk from baseline-to-12 months (p = .014, 95% CI [4.4, 39.1]) and a non-significant increase of 2.8 min/wk from 12-to-24 months (p = .756, 95% CI [-14.6, 20.2]). Next, among those receiving the CRM Incentive^+Support^ strategy, there was a significant mean increase of 45.9 min/wk of MVPA from baseline-to-24 months (p = .007, 95% CI [12.7, 79.1]; see Table 5). When examined across the respective data collection points, a significant mean increase of 38.2 min/wk of MVPA was observed from baseline-to-12 months (p = .023, 95% CI [4.9, 71.4]) and a non-significant mean increase of 7.7 min/wk from 12-to-24-month (p = .648, 95% CI [-25.5, 40.9]). For those in receipt of the CRM Incentive^Only^ strategy, Table 5 shows there was a non-significant mean increase of 12.2 min/wk of MVPA from baseline-to-24 months (p = .515, 95% CI [-24.6, 49.1]). Within this timeframe, the mean increase in MVPA was 0.32 min/wk from baseline-to-12 months (p = .987, 95% CI [-36.5, 37.2]) and 11.9 min/wk from 12-to-24-months (p = .526, 95% CI [-4.9, 48.8]). Finally, among those randomly allocated to the control condition, there was a non-significant mean increase of 15.4 min/wk of MVPA from baseline-to-24 months (p = .156, 95% CI [-5.9, 36.7]; see Table 5). Within this overall time period, there was an initial significant mean increase of 26.8 min/wk of MVPA from baseline-to-12 months (p = .014, 95% CI [5.5, 48.0]) followed by a reduction of 11.4 min/wk of MVPA from 12-to-24-months (p = .296, 95% CI [- 32.7, 9.9]). Fig 1 provides a graphical representation of these findings.

**Table 5:** Mean changes in total MVPA min/wk by data collection timepoints.

	Changes in total MVPA min/wk		
Group	Baseline-to-12 months	12-to-24 months	Baseline-to-24 months
All	21.7	2.8	24.5
Control	26.8	-11.4	15.4
CRM Incentive^Only^	0.32	11.9	12.2
CRM Incentive^+Support^	38.2	7.7	45.9

## Discussion

Identifying interventions to enhance the physical activity levels of typically inactive individuals with arthritis has important implications for improving their quality of life [3–5]. Yet for interventions to be successful, there needs to be a clear understanding of the theoretical constructs that drive change [55]. The longitudinal nature of the MOVE trial provided a unique opportunity to undertake CRM user profiling to better understand the socio-cognitive constructs predicting whether typically inactive individuals with arthritis reach/maintain different MVPA/wk thresholds over time and to explore the potential influence of two low-cost CRM strategies on their physical activity levels.

Taken together, the findings suggest that in the longer term, CRM strategies comprising incentives and ongoing supportive messaging have the potential to influence MVPA/wk thresholds among typically inactive individuals with arthritis. Notably, relative to the control group, receiving the Incentive^+Support^ CRM strategy was predictive of reaching/maintaining the WHO’s [30] recommended ≥150 min/wk of MVPA and the intermediate threshold of ≥45 min/wk at 24-months.

Importantly, the findings from our mixed effect models provide insights into mean change in total mins/wk of MVPA across time and reinforce the positive impact of using CRM-driven supportive messaging to encourage MVPA among individuals with arthritis. Specifically, the mean total minutes of MVPA for individuals receiving the Incentive^+Support^ CRM strategy increased significantly from baseline-to-24 months. Notably, while much of this increase appeared to have occurred in the first 12 months, it was maintained through the second year of the trial. This is a positive outcome given that prior research indicates sustained change in physical activity levels can be difficult to achieve in the longer term among individuals with arthritis [56–59]. It may be that the supportive messaging used in the Incentive^+Support^ condition helped to sustain engagement in physical activity by either keeping the importance of this behavior salient or offering tips for overcoming common barriers to physical activity. These findings warrant further research to determine ways to support and amplify this combined CRM approach. The lack of significant change in mean total MVPA min/wk among those receiving the Incentive^Only^ CRM strategy may be attributed to the potential undermining (or crowding out) effect of extrinsic incentives on intrinsic motivation, as suggested by self-determination theory literature [60]. Interestingly, the use of supportive messaging in the Incentive^+Support^ group may have protected against this by boosting intrinsic motivation. This interpretation aligns with recent research suggesting that crowding out can be mitigated when extrinsic incentives are used in combination with intrinsically motivating factors, including supportive emails and resources to enhance personal capacity for physical activity [61]. As noted above, this finding may explain why the mean total MVPA min/wk did not decline among participants receiving the Incentive^+Support^ CRM strategy. Finally, the excitement and publicity around the opening of the new PARC facility is a possible explanation for the significant increase in MVPA min/wk among the control group from baseline to 12 months.

Our findings also indicate that socio-cognitive drivers of physical activity can differ by MVPA threshold and across discrete timepoints, suggesting a need to consider developing CRM strategies on timepoint specific profiles. For example, the predictive role of intentions and action planning varied across timepoints for the intermediate MVPA threshold, such that baseline intentions were a significant predictor of participants with arthritis reaching/maintaining ≥45 min/wk of MVPA at 12-months post-baseline, whereas intentions measured at 12 months did not significantly predict meeting this threshold at 24-months post-baseline. Conversely, only action planning measured at 12-months post-baseline predicted this threshold at 24-months post-baseline. One potential explanation for these findings is that intentions to engage in physical activity may become crystalized into well-formed action plans with the passage of time, thus increasing the predictive utility of action planning. After all, action planning involves an individual conceptualizing when and where an intended behavior will be enacted [62]. Over time, cues from this detailed planning may become increasingly predictive of enactment, since associations can develop between the conceptual cues to perform the behavior and the behavior itself [63]. Importantly, research suggests that the relationship between action planning and physical activity is moderated by the strength of habit formation, such that those with well-established physical activity habits are less likely to rely on action planning relative to those with less formed habits [63]. This moderating effect may explain why action planning was not predictive of those reaching/maintaining the upper threshold of ≥150 min/wk of MVPA at either of our discrete timepoints.

In terms of anticipated regret, prior research suggests that this construct is predictive of exercise-related behavior in cross-sectional studies [44] and across short periods of time such as two weeks [64]. Our findings provide a more nuanced understanding of its motivational influence on physical activity across longer periods of time. Specifically, anticipated regret about being physically inactive was not associated with reaching/maintaining the upper ≥150 min/wk MVPA threshold, which is perhaps not surprising given that individuals achieving this upper threshold are less likely to consider themselves inactive and may therefore be less likely to experience anticipated regret about being physically inactive. In contrast, baseline anticipated regret scores were predictive of reaching or maintaining the intermediate ≥45 min/wk threshold at 12 months post-baseline, although scores from 12 months post-baseline were not predictive of reaching/maintaining this intermediate threshold 24 months post-baseline. This finding raises the question of whether individuals under-engaging in physical activity may become habituated to feelings of anticipated regret across time and thus become less responsive to them. The implications of this finding warrant further research, particularly in terms of how physical activity messages are framed to a cohort of individuals across time.

Individuals with arthritis are recommended to work towards the upper ≥150 min/wk MVPA threshold [65]. Our findings suggest that being involved in some form of organized physical activity may facilitate individuals with arthritis to achieve this recommendation, since organized physical activity was predictive of reaching/maintaining this level of physical activity at both 12- and 24-months post-baseline. These findings underscore the importance of CRM strategies in promoting engagement with such activities and point to the need for more research to explore the mechanisms underlying this relationship.

While the relationship between social connection and physical activity is under researched in the context of arthritis, prior research from community samples suggest a positive association between levels of social connection and physical activity such that those who are more socially isolated are more likely to have lower levels of physical activity [66–68]. Our findings provide only partial support for this association in the context of arthritis, with only baseline Friendship Scale scores (a measure of self-perceived social connection/isolation) predictive of reaching/maintaining ≥45min/wk of MVPA at 12 months post-baseline. It is possible that perceptions of being socially connected are important initial drivers of physical activity among those starting from a relatively low physical activity base but become less important over time.

Prior research is mixed on the relationship between gender and physical activity among individuals with arthritis, with some studies suggesting no gender differences and others suggesting physical activity is low among women [69, 70]. Our findings suggest that while females were significantly less likely than males to reach or maintain either the upper or intermediate MVPA thresholds at 12 months post-baseline, gender was no longer predictive of these outcomes at 24 months post-baseline.

### Future research

Given the CRM strategies provided to the Incentive^+Support^ group were general in nature (i.e., not tailored to a specific individual or disease), future research is needed to examine whether additional minutes of MVPA/wk could accrue through incorporating disease-specific elements into the CRM program content. Further research is also needed to explore whether additional CRM elements could be introduced at 12 months post-baseline to boost mean minutes of MVPA/wk in the subsequent year. At a strategic level, exploration of the optimal frequency of delivering CRM program content to system recipients would also be valuable; too much could be perceived as annoying while too little could lessen the effectiveness of the overarching CRM strategy.

### Limitations

The MOVE trial from which the data from the current study were drawn, and indeed the current study, are not without limitations. First, physical activity was measured by self-report at each timepoint rather than device-based measures, such as accelerometers. As such, recall bias and social desirability bias cannot be ruled out. Second, we drew on a subsample of the randomized participants in the MOVE trial who self-reported having arthritis, but we were not able to confirm the type or severity of their arthritis. Third, we applied the missing at random assumption and replaced missing values with LOCF. This may have led to the underestimation or overestimation of some of the associations identified.

## Conclusion

Our findings show that the use of a CRM strategy delivering incentives and supportive messaging increases mean total min/wk of MVPA among individuals with arthritis. Such a strategy is also helpful in encouraging individuals with arthritis to reach or maintain both the WHO [30] and an intermediate MVPA threshold 24 months post-baseline. Moreover, the findings suggest socio-cognitive profiles may be useful in guiding how elements of the CRM strategy are focused and framed and are therefore of relevance to teams responsible for overseeing multi-purpose leisure, aquatic, and fitness centers. Specifically, since the socio-cognitive factors influencing physical activity in the current study varied based on both MVPA threshold and time, center management should consider tailoring their CRM strategies to the socio-cognitive profile of the recipients of those strategies. Taken together, the findings contribute to the emerging literature around using CRM systems to foster health-related benefits among recipients.

## Supporting information

S1 Appendix Description of measures used. (DOCX)

S1 Dataset (DTA)
