# Supplementary material for: Effects of customer relationship management (CRM) strategies and socio-cognitive constructs on the physical activity of individuals with arthritis over time
Source: PLoS One. 2023 Oct 10;18(10):e0292692. doi: 10.1371/journal.pone.0292692 (PMC10564229; doi:10.1371/journal.pone.0292692)
Supplement: S1 Appendix — (DOCX) [file pone.0292692.s001.docx]

S1 Appendix: Description of the measures used

| Construct | Description |
| --- | --- |
| Exercise Recreation and Sport Survey (ERASS) | ERASS [39] assesses the frequency and duration of organized and non-organized leisure activities over a specified time period, which in this study was the past 12 months and the past two weeks. The data gathered from ERASS was used to calculate the total min/wk of MVPA, including two MVPA thresholds: the WHO’s [30] recommended ≥150 min/wk of MVPA threshold and our intermediate threshold of ≥45 min/wk of MVPA. |
| The constructs assessing social cognitive decision making around physical activity were derived from the Theory of Planned Behavior framework and its extensions [41- 42]. All items followed the measurement guidelines proposed by Fishbein and Ajzen [41-42]. Specifically, the action, target, context, and time of the behavioural criterion (i.e., regular physical activity) were compatible across all items. Participants rated their agreement on a 5-point Likert-type scale where 1=strongly disagree and 5=strongly agree. | |
| Intention | Single item measure assessing intention to exercise regularly. Specifically, “I intend to exercise regularly”. |
| Attitude | Single item measure of attitude towards engaging in physical activity [40-42]. Specifically, “For me, exercising regularly would be pleasant”. |
| Subjective norm rom | Single item measure assessing the degree to which participants agreed that others who would approve if they exercised regularly [43]. Specifically, “Most people who are important to me would approve if I exercised regularly”. |
| Self-efficacy | Single item measure of participants perceived ability to exercise if they wanted to [43]. Specifically, “I believe I have the ability to exercise regularly if I wanted to.” |
| Anticipated regret | Single item measure of the degree to which participants would feel anticipated regret if they did not exercise regularly [44]. Specifically, “I think I would regret it if I did not exercise regularly”. |
| Action planning | Single item measure assessing the degree to which participants felt they had developed an action plan for physical activity [45]. Specifically, “I have made a plan about where to exercise regularly”. |
| Global self-rated mental health | A single item was used to capture self-rated mental health. Specifically, participants responded to the question “In general, would you say your physical health is…” using a 5-point Likert-type scale, where 1=excellent and 5=poor [46]. |
| Global self-rated physical health | A single item was used to capture self-rated mental health. Specifically, participants responded to the question “In general, would you say your mental health is…” using a 5-point Likert-type scale, where 1=excellent and 5=poor [47]. |
| Friendship scale (6-item scale] | The 6-item friendship scale provides a self-report measure of social support. Each item is assessed on a 5-point Likert-type scale where 1=Almost always and 5=Not at all. The scale has demonstrated reliability (Cronbach α=0.83) and discriminate validity [49]. |
| Two items from the Australian Unity Wellbeing Index were used to assess participant feelings of being supported and not socially isolated (50). Each item was assessed using an 11-point Likert-type scale where 0=very dissatisfied, 5=neutral, 10=very satisfied. These items have demonstrated construct validity with respect to other measures of life satisfaction and community wellbeing [50]. | |
| Feeling part of the community | Participants rated their sense community connectedness on a single item that read “How satisfied are you with feeling a part of your community?” [50]. |
| Sense of trust in others (most people can be trusted) | Participants rated their feeling of trust in others on a single item that read “How satisfied are you that most people can be trusted?” [50]. |
